# Reproductive biology of an endangered lithophytic shrub and implications for its conservation

**DOI:** 10.1186/s12870-022-03466-3

**Published:** 2022-02-22

**Authors:** Yuan-Mi Wu, Xue-Li Shen, Ling Tong, Feng-Wei Lei, Xiao-Fei Xia, Xian-Yun Mu, Zhi-Xiang Zhang

**Affiliations:** 1 Laboratory of Systematic Evolution and Biogeography of Woody Plants, School of Ecology and Nature Conservation, Beijing Forestry University, Beijing, 100083 China; 2 Beijing Museum of Natural History, Beijing, 100050 China

**Keywords:** *Lonicera oblata*, Endangered plant, Floral syndrome, Mixed mating system, Pollination, Pollen limitation, Conservation

## Abstract

**Background:**

Plants in cliff habitats may evolve specific reproductive strategies to cope with harsh environments, and unraveling these reproductive characteristics can improve our understanding of survival strategies and lithophyte evolution. This understanding is especially important for efforts to protect rare and endemic plants. Here, we investigated the reproductive biology of *Lonicera oblata*, an endangered lithophytic shrub that is scattered in highly fragmented and isolated cliff habitats of the Taihang and Yan mountains in North China.

**Results:**

Flowers of *L. oblata* are herkogamous and protandrous, characteristics that can prevent autogamy at the single-flower level, and insects are necessary for pollination. The outcrossing index, pollen/ovule ratio, and the results of hand pollination were measured and all revealed a mixed mating system for *L. oblata*, that combines cross-fertilization and partial self-fertilization. The floral traits of *L. oblata* of zygomorphic and brightly yellowish corolla, heavy fragrance, and rich nectar, suggest an entomophilous pollination system. Sweat bees were observed as the most effective pollinators but their visiting frequencies were not high. Pollen limitation may limit the reproductive success of *L. oblata*.

**Conclusions:**

We determined the reproductive characteristics of *L. oblata*, a critically endangered species endemic to cliffs in North China, providing insight into its endangerment and suggesting conservation strategies. *L. oblata* has highly pollinator-dependent self-fertilization as part of a mixed mating system. Floral features such as low-flowering synchrony, asynchronous anthers dehiscence, and high duration of stigma receptivity, improve pollination efficiency in the case of low pollinator service. Our work provides reference information to understand the survival strategies and conservation of *L. oblata* and other lithophytes.

**Supplementary Information:**

The online version contains supplementary material available at 10.1186/s12870-022-03466-3.

## Background

Plant rarity is related to evolutionary and ecological processes, which are influenced by life-history features, interactions with other species and environmental conditions, and anthropogenic effects [1–4]. Reproduction is an essential and relatively fragile stage in the life cycle of plants, and it is key to evolution [5]. Therefore, understanding reproductive characteristics, including pollination ecology and breeding system, is required for the identification of specific threats to rare plants, particularly for species that live in harsh environments where pollination is limited [3].

Cliff habitats are among those habitats where pollinators are sparse or uncertain, and these habitats are reservoirs of relict biodiversity with a large number of rare and endemic plants [6, 7]. Cliff-dwelling plants are often subjected to harsh environmental pressures and ecological constraints (e.g., moisture shortage, thin soil, poor organic matter, and high alkaline soil). These plants also typically experience extreme and harsh climate (e.g., low temperature, high solar radiation, and strong wind), which may result in stronger pressure on pollination and seed setting [6–8]. Cliffs are often surrounded by forests, resulting in a natural fragmented and isolated landscape of “ecological islands” [6, 9]. Plants that grow in such isolated islands on cliffs are less likely to disperse pollen and seed over long distances [7]. Cliff habitats are sensitive to climate change, which can disrupt the overlap in seasonal timing of flower production and pollinator activity to further reduce pollination [10–12]. The reproductive success of these lithophytes can be reduced by both uncertain pollinator service and low pollen output [3, 7, 9]. Studies of reproductive characteristics can improve our understanding of the evolutionary process and survival strategies of these rare and endemic lithophytes, yet there have been few studies, at least partially due to the difficulty of working on cliffs [13].

The area containing the Taihang and Yan mountains is one of 35 priority areas for biodiversity conservation in China (http://www.mee.gov.cn/gkml/hbb/bgg/201601/t20160105_321061.htm). There are the highest plant diversity and a high rate of endemism in North China, with a variety of impressive cliff habitats. This area is also home to a plethora of rare and endemic lithophytic plant species, such as *Clematis acerifolia* Maxim. (Ranunculaceae), *Corydalis fangshanensis* W. T. Wang ex S. Y. He (Papaveraceae), *Opisthopappus taihangensis* (Y. Ling) C. Shih (Asteraceae), *Oresitrophe rupifraga* Bunge (Saxifragaceae), and *Taihangia rupestris* T. T. Yu & C. L. Li (Rosaceae) [14]. However, little is known about the reproductive characteristics and genetic patterns of these endemic plant species [15, 16].

**Fig. 1:**
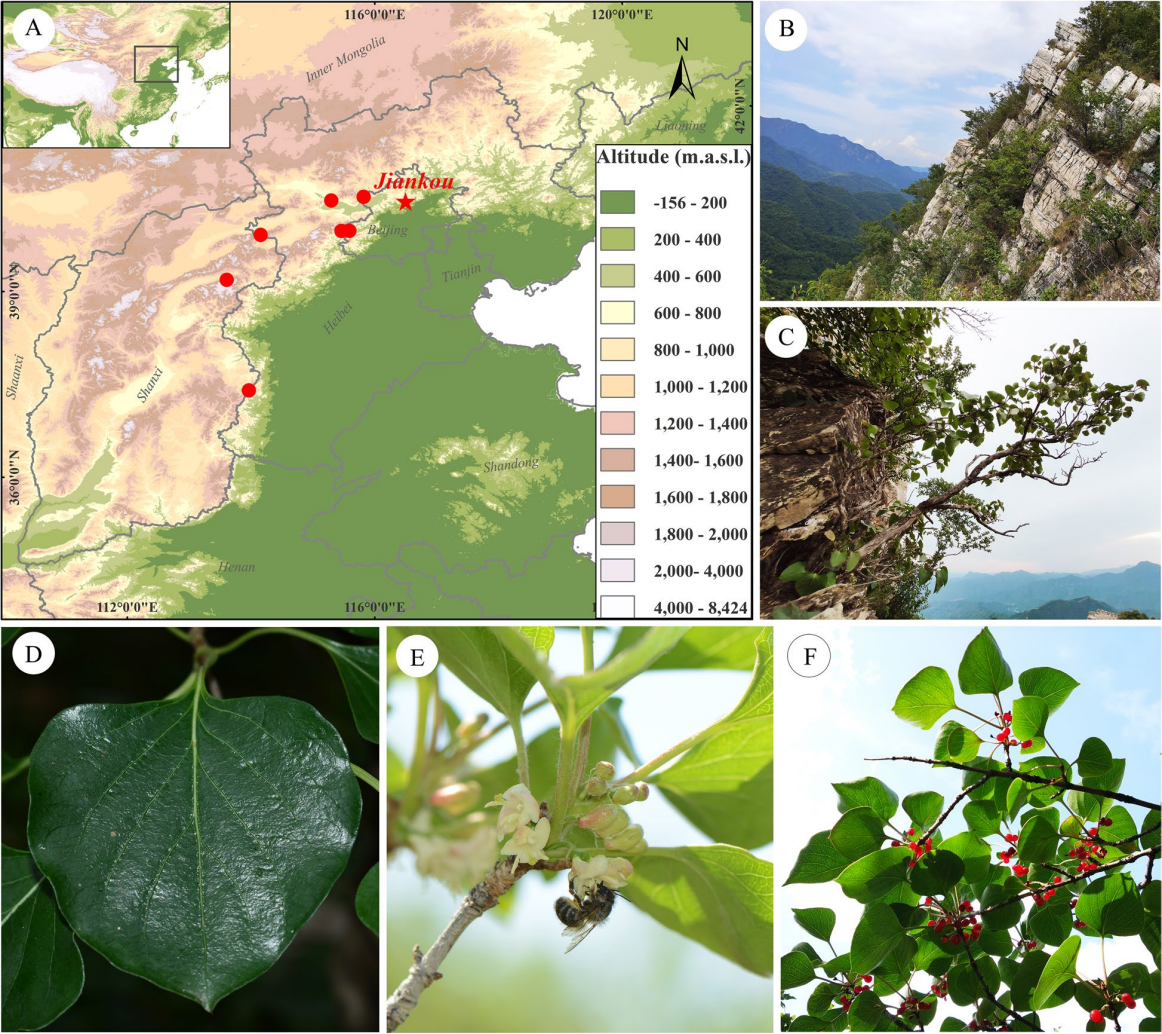
Photos of *Lonicera oblata* in the wild and its distribution. **A** Distribution sites of *L. oblata*. **B** Habitat of *L. oblata*. **C** Individual of *L. oblata*. **D** Single leaf of *L. oblata*. **E** Flowers of *L. oblata*. **F** Fruits of *L. oblata.* A map of China was obtained from the Data Center for Resources and Environmental Sciences, Chinese Academy of Sciences (RESDC, http://www.resdc.cn)

*Lonicera oblata* K.S. Hao ex P.S. Hsu & H.J. Wang is a deciduous shrub that is endemic to this area. It is listed as a second-level plant on the National Key Protected Wild Plants of China (http://www.forestry.gov.cn/main/5461/20210908/162515850572900.html). Thirteen years of fieldwork allowed the identification of eight highly fragmented populations growing in limestone habitats at an altitude of about 1000 m (Fig. 1A). Individuals were found growing in small cracks and shallow-soiled ledges near or on the top of exposed steep limestone cliffs (Fig. 1B–C) [17]. Human activities (e.g., logging, mining, overgrazing, and tourism) may have provided pressure on the survival of *L. oblata*. This species may also be sensitive to climate change, and future climate warming could further decrease the availability of suitable habitats for this lithophytic shrub [18]. Therefore, study of this endangered species is representative of plants endemic to limestone cliff habitats in the Taihang and Yan mountains.

In this study, we investigated the reproductive biology of *L. oblata* and identified its potential threats. To do this, we performed a long-term field study of the pollination ecology and breeding system of this endangered species. Our study of the reproductive system of *L. oblata* was designed to address the following questions: (1) What are the reproductive characteristics of *L. oblata*? (2) How do floral traits associate with pollinators and influence its pollination success? (3) How does the breeding system of *L. oblata* promote its reproductive success? Based on the results, several conservation strategies were proposed.

## Results

### Phenology, floral morphology, and blossom development

*Lonicera oblata* is an early-spring flowering species. With increasing temperature in March, flower buds of *L. oblata* started to develop and differentiate. Leaf buds started to develop in April, and flowers opened from the end of April to late-May, peaking in the first week of May. After pollination, ovaries started to dilate in early June and fruits turned red after ripening in the middle of July. The leaves started to drop in the middle of September.

*Lonicera oblata* has paired-flower inflorescences and its total pedicels cluster in the proximal leaf axils of young branchlets. The flowers are large (9.15 ± 1.80 mm in length) and bright yellowish, typically with zygomorphic and two-lipped corolla. The sympetalous corolla forms a tube about 4.03 ± 0.78 mm long, and there is a unilaterally saccular bulge in the lower part, which is lined with nectary secretory tissue and produces a large amount of nectar with heavy-sweat fragrance. The produced nectar often accumulates at the base of the corolla tube. The length and width of the floral tube in the mature flower in full-dehiscence stage (described below) are 4.03 ± 0.78 mm (Mean ± SD, consistent with the following) and 2.13 ± 0.48 mm, respectively. There are five stamens in each flower, with an average length of 9.43 ± 1.57 mm. This is significantly longer than the average length of stigma (8.00 ± 0.97 mm) (Student’s t-test, t = 9.229, *P* < 0.0005). The detailed floral morphological characteristics are presented in Table 1.

**Table 1:** Floral morphology of *Lonicera oblata*

Characters	Sample size	Range	Mean ± SD
Pedicel length (mm)	30	2.61–18.10	9.96 ± 3.94
Flower length (mm)	60	11.67–32.74	21.37 ± 5.19
Corolla length (mm)	60	5.71–13.47	9.15 ± 1.80
Corolla diameter (mm)	60	5.30–12.38	9.21 ± 1.75
Tube length (mm)	60	2.52–5.45	4.03 ± 0.78
Tube width (mm)	60	0.62–3.24	2.13 ± 0.48
Stamen height (mm)	300	5.87–13.72	9.43 ± 1.57
Stigma height (mm)	60	5.85–9.80	8.00 ± 0.97
Number of pollen grains (P)	36	12,700–25,840	18,902.22 ± 3178.38
Number of ovules (O)	36	4–11	8.33 ± 1.22
Ratio of pollen grains to ovules (P/O)	36	1155–4710	2353.94 ± 686.74

**Fig. 2:**
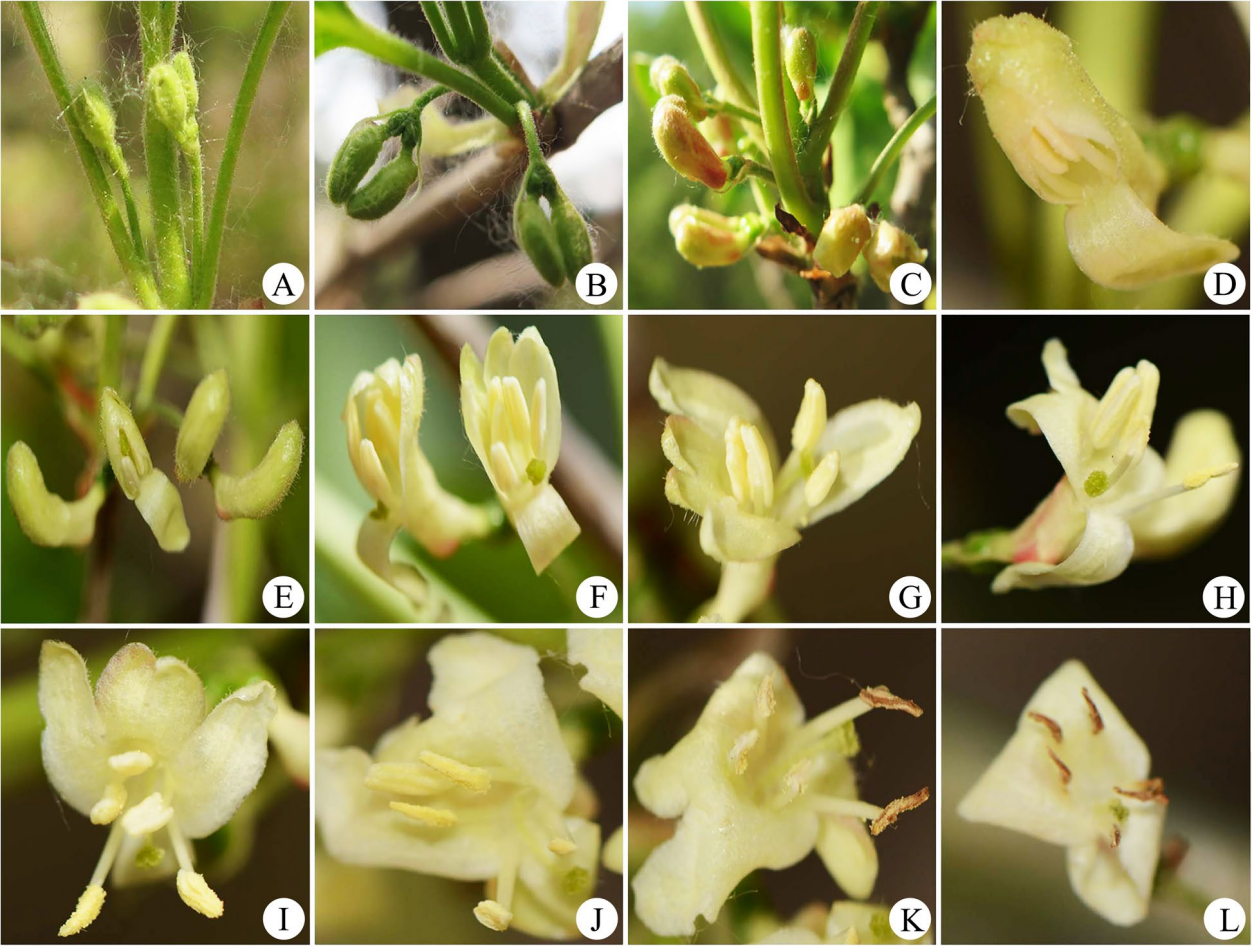
Flowering phenology of *Lonicera oblata*. **A–C** Flower bud stage. **D–F** Pre-dehiscence stage. **G** Initiating dehiscence stage. **H–J** Full-dehiscence stage. **K** Post-dehiscence stage. **L** Flower wilting stage

**Fig. 3:**
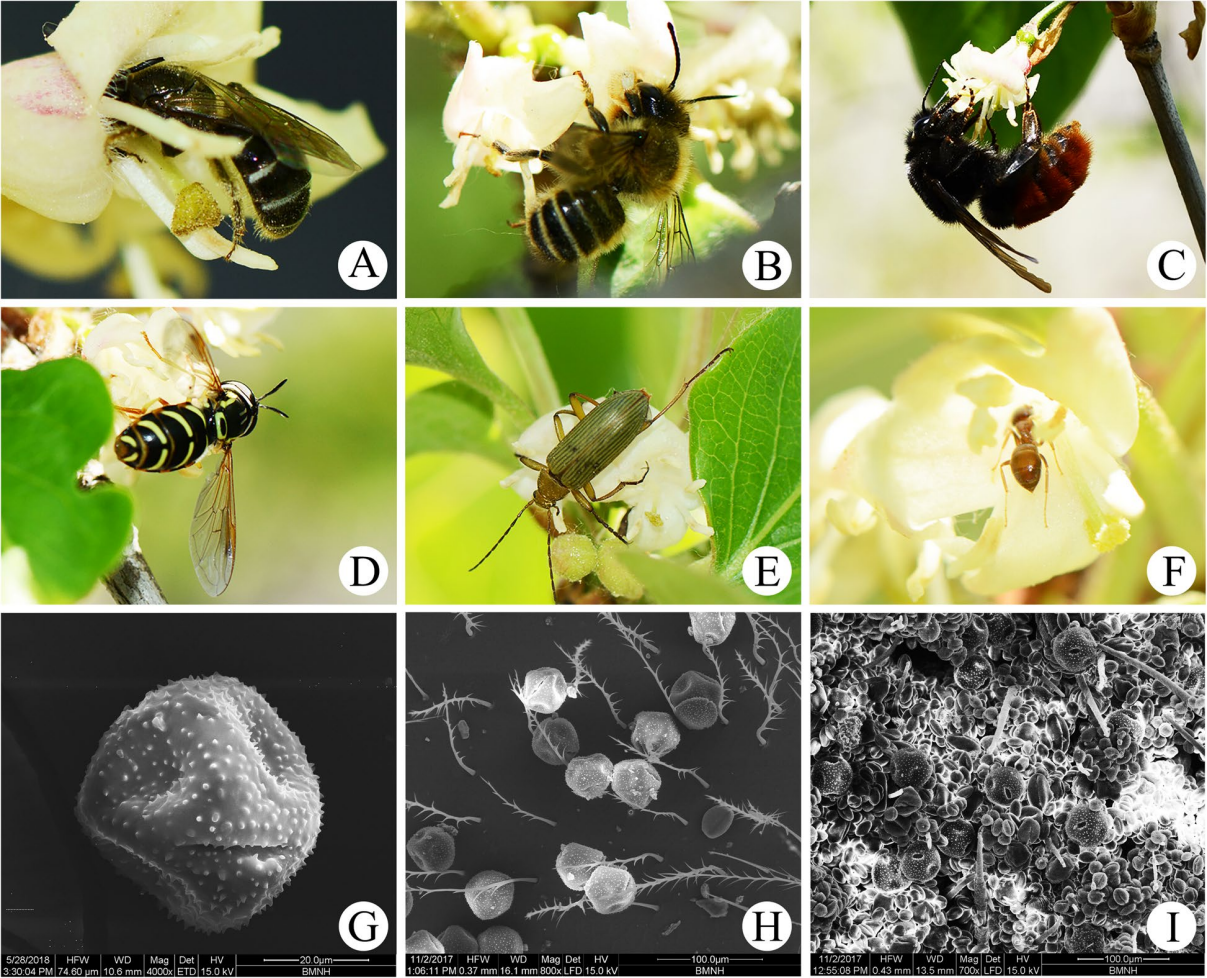
Floral visitors and pollen of *Lonicera oblata*. **A** *Lasioglossum* sp. **B** *Megachile* sp. **C** *Bombus* sp. **D** Syrphidae sp. **E** Tenebrionidae sp. **F** Myrmicinae sp. **G** Pollen grains of *L. oblata* under scanning electron microscope (SEM) **H** Pollen grains on *Lasioglossum* sp. **I** Pollen grains on *Bombus* sp.

**Fig. 4:**
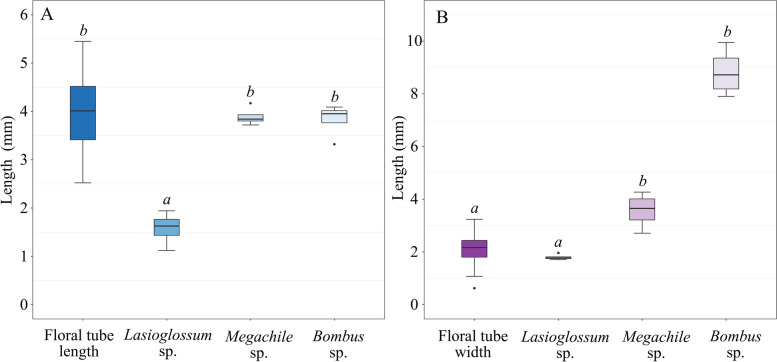
Length and width of the floral tube of *Lonicera oblata* and its relationship to that of the three pollinators. **A** Floral tube length and tongue length of the main floral visitors. **B** Floral tube width and body width of the main floral visitors. Different italic lowercase letters above each box indicate significant differences at *P* < 0.0005

**Fig. 5:**
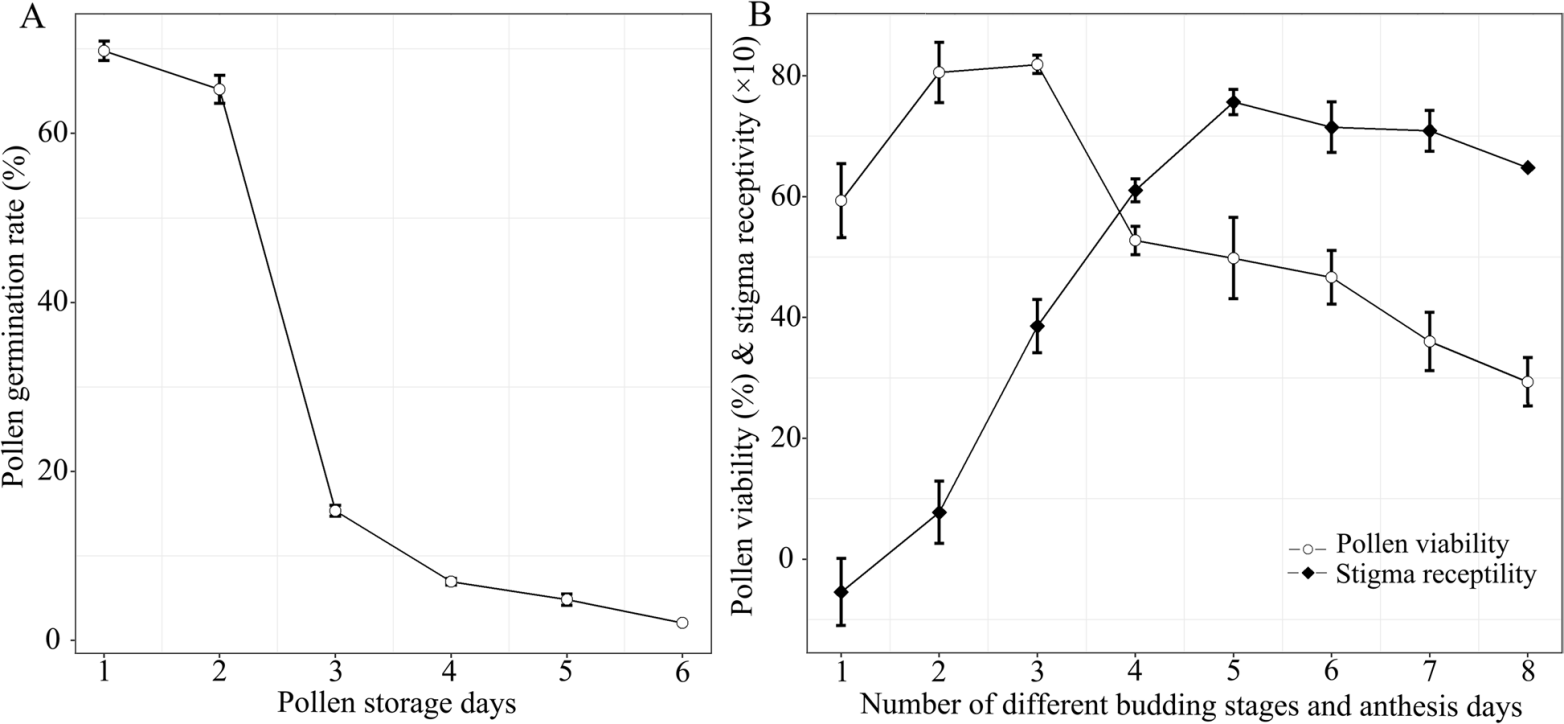
The pollen lifespan (**A**) and changes in pollen viability and stigma receptivity (**B**) of *Lonicera oblata*. In the abscissa of plot **B**, numbers 1–3 represent three budding stages with bud lengths of 5.36 ± 0.51 mm, 6.81 ± 0.59 mm, and 8.63 ± 0.62 mm, respectively; numbers 4–8 represent different flowering days (1–5) with anther indehiscence, dehiscence of the two front anthers, dehiscence of all five anthers, brown anthers, and wilting stamens, respectively

**Fig. 6:**
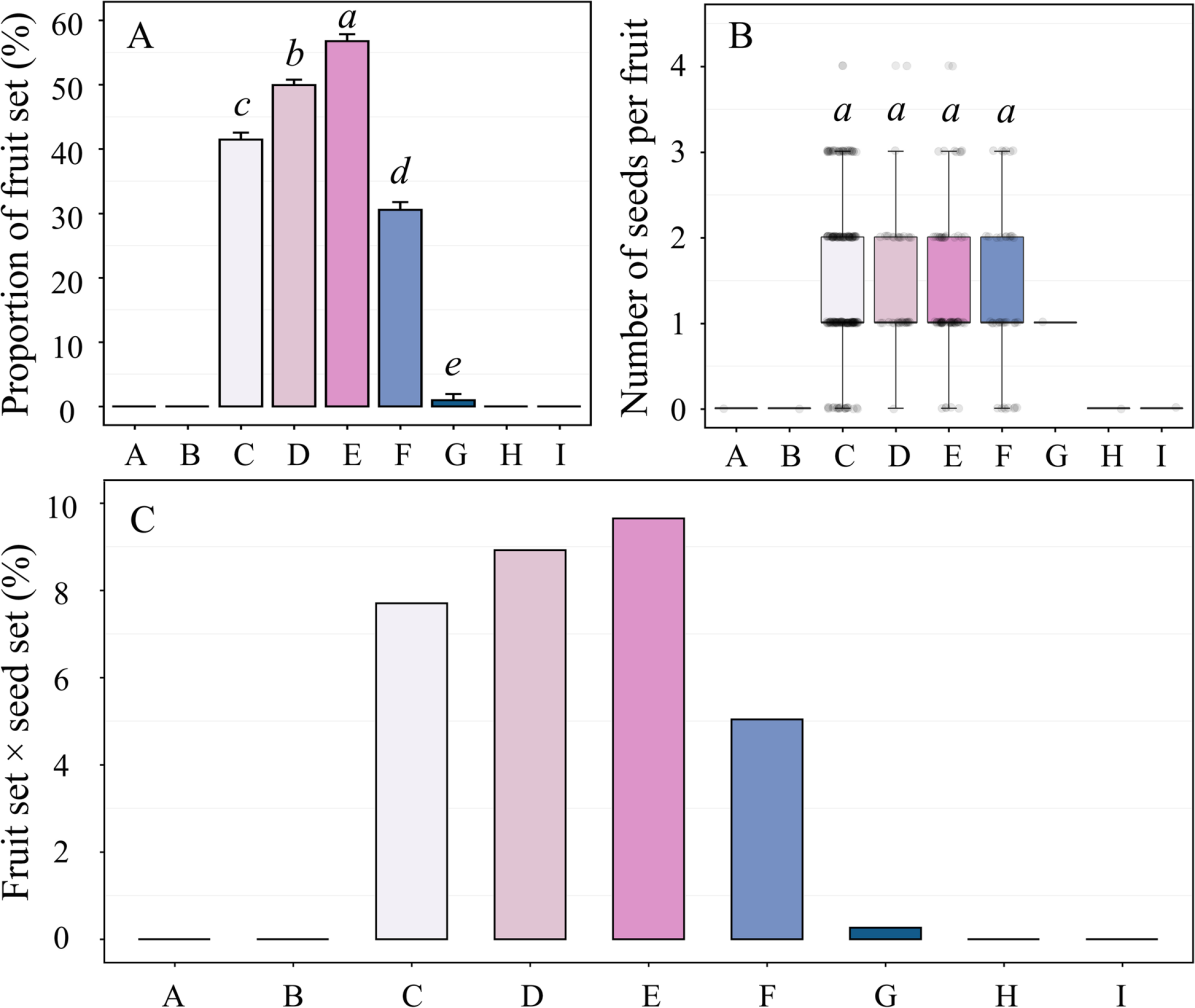
Proportion of fruit set **(A)**, number of seeds per fruit **(B)**, and product of the average fruit set and the average seed set **(C)** for *Lonicera oblata* subjected to different pollination treatments. A: Isolation animal pollination; B: Anemophilous pollination; C: Natural pollination; D: Pollinator-mediated cross-pollination; E: Xenogamous pollination; F: Geitonogamous pollination; G: Manual self-pollination; H: Spontaneous self-pollination; I: Apomixes. Different italic lowercase letters above each box in plots **A** and **B** indicate significant differences at *P* < 0.05

The flowering time of *L. oblata* in the studied population was approximately 40 days in duration, and a single flower opened for about 8 days. The floral phenology of a single flower can be divided into six stages: (1) flower bud stage: the buds grew gradually to a length of 13.89 ± 0.97 mm, and the base of the petal gradually turned red (Fig. 2A–C); (2) pre-dehiscence stage: one petal at the downside opened first and reflexed, then the two anthers close to the open petal exserted. The stigma extended along the open petal, and the other petals expanded successively (Fig. 2D–F); (3) initiating dehiscence stage: two front anthers began to dehisce, which lasted for about a half-day (Fig. 2G); (4) full-dehiscence stage: the other four petals opened and reflexed, the remaining three anthers gradually dehisced, and the mucilage on the stigma gradually increased (Fig. 2H–J); (5) post-dehiscence stage: pollen of the two front anthers was dispersed completely and the anthers turned brown (Fig. 2K); (6) flower wilting stage: all anthers and stigma turned brown, and the flower finally wilted (Fig. 2L). The duration of flowering in a single flower was closely related to the external environment. For individuals growing on high clifftops, once the anthers dehisced, the pollen was completely blown away by wind the next day, but the anthers of individuals growing under shrublands lasted for about 2 days.

### Floral visitors and their behavior

Several species of insects were recorded visiting the flowers of *L. oblata*, including sweat bees (*Lasioglossum* sp., Halictidae, Hymenoptera, Fig. 3A), megachilid bees (*Megachile* sp., Megachilidae, Hymenoptera, Fig. 3B), bumblebees (*Bombus* sp., Apidae, Hymenoptera, Fig. 3C), hoverflies (Syrphidae sp., Diptera, Fig. 3D), darkling beetles (Tenebrionidae sp., Coleoptera, Fig. 3E), and ants (Myrmicinae sp., Hymenoptera, Fig. 3F). These insects can be classified into two categories according to their flower-visiting behavior. The first category includes insects that are usually small in size with a glabrous body, including hoverflies, darkling beetles, and ants. These insects gnawed filaments, petals, and stigmas or made a small hole at the base of the corolla tube to directly obtain nectar, thus making little contribution to pollination. The second category includes insects such as sweat bees, megachilid bees, and bumblebees. These insects may be more effective pollinators, and were observed landing on the corolla or entering the corolla tube to obtain nectar. During foraging, the bodies of these visiting insects contacted the stigma and the anthers repeatedly, and these insects moved frequently over the clustered flowers.

Sweat bees, megachilid bees, and bumblebees were the most frequent visitors to *L. oblata*, but there was significant variation in the visitation frequency and duration time. The visitation frequency of sweat bees (5.25 ± 1.64 visits/flower/h) was significantly higher than those of megachilid bees (1.81 ± 0.82 visits/flower/h) and bumblebees (0.08 ± 0.14 visits/flower/h) (one-way ANOVA, F_(2,9)_ = 18.40, *P* = 0.003, Table 2). Sweat bees perched on the downside of two filaments that were first to elongate and mature to collect pollen with their abdomen and legs, and finally crept into the floral tube to obtain nectar. During this process, their abdomens touched the stigma repeatedly, promoting effective pollination (Fig. 3A; Video S1). The sweat bees spent significantly longer time on flowers (15.29 ± 0.58 s) than the megachilid bees (2.00 ± 0.70 s) and the bumblebees (1.92 ± 0.55 s) (one-way ANOVA, F_(2,9)_ = 625.59, *P* < 0.0005, Table 2). A large number of pollen grains of *L. oblata* (Fig. 3G) were detected on the bodies of *Lasioglossum* sp. by using scanning electron microscopy (SEM) (Fig. 3H), while only a small amount of pollen grains were found on *Bombus* sp. bodies (Fig. 3I).

**Table 2:** Floral visitors and their body sizes, visiting frequencies and duration time on *Lonicera oblata* (Mean ± SD)

Floral visitor	*Lasioglossum* sp.	*Megachile* sp.	*Bombus* sp.
Body length (mm)	6.33 ± 0.75*a*	10.45 ± 1.07*b*	21.34 ± 0.42*c*
Body width (mm)	1.80 ± 0.11*a*	3.57 ± 0.68*b*	8.82 ± 0.92*c*
Tongue length (mm)	1.58 ± 0.35*a*	3.64 ± 0.56*b*	3.83 ± 0.35*b*
Tongue & head length (mm)	2.35 ± 0.13*a*	7.35 ± 0.40*b*	9.14 ± 1.12*c*
Head width (mm)	1.74 ± 0.06*a*	3.40 ± 0.47*b*	5.94 ± 0.43*b*
Time on a single flower (s)	15.29 ± 0.58*a*	2.00 ± 0.70*b*	1.92 ± 0.55*b*
Visit frequency (visits/flower/h)	5.25 ± 1.64*a*	1.81 ± 0.82*b*	0.08 ± 0.14*b*
Body parts with pollen attached	all parts of body, mainly abdomen and legs	tongue, legs, abdomen	tongue, legs

Note: different italic lowercase letters following numbers indicate significant differences at *P* < 0.05

The body sizes of the three main floral visitors (i.e., sweat bees, megachilid bees, and bumblebees) were compared to the morphological characteristics of flowers. The tongue length of sweat bees (1.58 ± 0.35 mm) is significantly shorter than the corolla tube length of *L. oblata* (4.03 ± 0.78 mm) (one-way ANOVA, F_(3,67)_ = 167.33, *P* < 0.0005, Fig. 4A and Table 2). The tongues of megachilid bees and bumblebees were much longer (3.64 ± 0.56 mm and 3.83 ± 0.35 mm, respectively), and not significantly different from the average length of the corolla tube (one-way ANOVA, F_(3,48)_ = 37.40, *P* < 0.0005, Fig. 4A and Table 2). The corolla tube width of *L. oblata* (2.13 ± 0.48 mm) was slightly longer than the body width of sweat bees (1.80 ± 0.11 mm), but it was significantly narrower than those of megachilid bees (3.57 ± 0.68 mm) and bumblebees (8.82 ± 0.92 mm) (one-way ANOVA, F_(3,67)_ = 167.33, *P* < 0.0005, Fig. 4B and Table 2).

### Pollen lifespan, pollen viability, and stigma receptivity

Pollen germination was higher in the first two stages (69.78 ± 1.67% and 65.23 ± 2.85%), drastically decreased to 15.34 ± 1.13% at the third stage, and was extremely low in the fourth stage (Fig. 5A), suggesting a three-day lifespan of pollen grains. Pollen viability was highest (81.87 ± 3.02%) at the third stage, but decreased dramatically (52.74 ± 4.72%) at the first day of corolla opening (Fig. 5B and Table S1). Stigmas exhibited low receptivity during the budding stages. This gradually increased after corolla opening for a high level of receptivity (6.10 ± 0.37–7.57 ± 0.42) until the end of flowering (Fig. 5B and Table S1). Although there was a short period of overlap of pollen viability and stigma receptivity, the peak stage of stigma receptivity was much later than that of pollen viability.

### Mating system

The average diameter of the opening corolla was 9.21 ± 1.75 mm (Table 1), which was longer than 6 mm, so this was scored as 3 following the criterion of Dafni [19]. The stamens matured first (Fig. 5B), which was consistent with dichogamy, so this was scored as 1. The heights of stamen and stigma were 9.43 ± 1.57 mm and 8.00 ± 0.97 mm (Table 1), respectively, forming spatial isolation (Student’s t-test, t = 9.229, *P* < 0.0005), for a score of 1. Therefore, the outcrossing index (OCI) was considered 5, indicating a breeding system of *L. oblata* was outcrossing with partial self-compatibility and requiring pollinators. The number of pollen grains, ovules, and the ratio of pollen grains to ovules (P/O) were 18,902.22 ± 3178.38, 8.33 ± 1.22, and 2353.94 ± 686.74, respectively (Table 1). According to the criterion of Cruden [20], the breeding system was facultative xenogamy, which is consistent with the OCI classification.

Abortion from ovaries of both the emasculated flowers covered with waxed paper and mesh bags with 80 mesh filter was observed, and no fruit production was observed in the non-manipulated but bagged flowers (Fig. 6A and Table S2). Treatment settings of no fruit were removed from further analysis. We did statistical analysis of the other five pollination treatments and the proportion of fruit set varied significantly (one-way ANOVA, F_(4,15)_ = 440.992, *P* < 0.0005, Fig. 6A and Table S2): xenogamous pollination (56.76 ± 2.13%) > pollinator-mediated cross-pollination (49.91 ± 1.72%) > natural pollination (41.47 ± 2.17%) > geitonogamous pollination (30.55 ± 2.41%) > manual self-pollination (0.96 ± 1.92%).

Excluding the treatments without seed set and manual self-pollination (only one seed), seed production of the other four treatments was also performed but there was no significant difference between them (*K* = 2.213, *df* = 3, *P* = 0.529, Fig.6B and Table S2). Furthermore, we calculated the product of the average fruit set and the average seed set under different treatments, the values of them were very low, not exceeding 10% (Fig.6C and Table S2).

## Discussion

### Floral syndromes and effective pollinator

Floral syndromes (i.e., pollination syndromes) are suites of integrated floral traits (e.g., morphology, color, scent, and rewards) that may reflect convergent adaptations of flowers pollinated by specific types of pollen vectors [21]. Floral traits have been associated with different pollination syndromes [21], and they may evolve to match the morphology and behavior of pollinators [22]. The floral syndromes of *L. oblata* (e.g., bisexual and zygomorphic flowers, tubular and bilabiate corolla, and brightly yellowish tepals) indicate that its pollination system should be animal-mediated cross-pollination. In addition, there is a unilaterally saccular bulge in the lower part of the floral tube, which is lined with nectary secretory tissue and produces a large amount of nectar. The abundant nectar has heavy-sweat fragrance and provides precious forage for flower-visiting insects, especially in early spring. Our flower-visiting observation and hand pollination treatments support that the pollination system of *L. oblata* is animal-mediated cross-pollination.

The main floral visitors of *L. oblata* are hymenopteran species (i.e., sweat bees, megachilid bees, and bumblebees). The flowers of *L. oblata* have characteristics that are associated with bee pollination [23], such as short tubular corollas and small amounts of concentrated nectar. Interestingly, the two stamens that were the first to ripen and elongate are served as a landing platform for sweat bees (Fig. 3A). The sweat bees have short tongues (Fig. 4A) and slender bodies (Fig. 4B), so they enter the corolla tube to feed on nectar. Because the stigma of *L. oblata* is located mostly below the anthers (i.e., reverse herkogamy [24]), the abdomens of sweat bees could contact the stigma repeatedly for effective pollination during foraging (Video S1). In contrast, megachilid bees and bumblebees have large-sized bodies (Fig. 4A) and longer tongues (Fig. 4B), so they are not able to enter the corolla tube. Instead, these visitors landed on the corolla lips and sucked nectar directly with their long beaks (Fig. 3B–C). Therefore, sweat bees are suggested the most effective pollinators of *L. oblata*.

### Floral longevity and pollen limitation

Extended floral longevity may be an evolutionary strategy employed to overcome sparse or unpredictable pollinator service by increasing the amount of own pollen exported and foreign pollen imported [25, 26]. The five stamens of *L. oblata* elongate and mature asynchronously, and this gradual dehiscence of anthers may prolong the duration of pollen presentation [27, 28]. Extension of the pollen presentation could increase the time of pollen collection for pollinator [29]. The stigma of *L. oblata* was observed to maintain high receptivity for a relatively long period after corolla opening (Fig. 5B). This may further increase the chance of being pollinated.

Pollen limitation of female fecundity is a common phenomenon among flowering plants, particularly for animal-pollinated species [30]. We observed a reduction of product of fruit set and seed set for flowers with natural pollination (7.70%, Fig. 6C and Table S2) compared to flowers that were manually hand cross-pollinated (pollen supplementation, 9.65%, Fig. 6C and Table S2), suggesting pollen limitation. Because there was no significant difference in seed set between natural pollination and hand cross-pollination, pollen limitation may be mainly due to insufficient pollen vectors, inadequate pollen availability, and inefficient pollen transfer. First, the flowers of *L. oblata* bloom in early spring, when there are few floral resources to attract potential pollinators. The low temperature in the early spring in North China may also decrease the diversity and activity of pollinators [31], especially in cliff habitats [7, 9]. Second, the extremely low numbers of populations and individuals of *L. oblata* limit the effective pollination among individuals, further reducing the potential for cross fertilization among populations. Only eight populations of *L. oblata* have been found, and these plant habitats are highly fragmented. The survival of *L. oblata* is under great pressure from other plants with similar ecological niche (unpublished data), harsh climate and environment, and anthropogenic activities [18]. Limited pollination may affect the genetic composition of intra- and inter-populations, and this might decrease the long-term survival of *L. oblata*.

### Pollinator-dependent mixed mating system

Very few plants are absolutely self-crossing or outcrossing in nature. Instead, most plants exhibit a mixed mating system that combines self-fertilization and cross-fertilization for reproductive success [32], especially as a strategy to cope with unstable environments [33]. In this study, the outcrossing index (OCI) and ratio of pollen grains to ovules (P/O) as well as the results of hand pollination treatments, suggested that *L. oblata* is self-compatible and cross-fertile, and its breeding system is mixed mating. There was no fruit production in the emasculated flowers covered with waxed paper and mesh bags (Fig. 6A and Table S2), suggesting that apomixes and anemophilous pollination may not occur in *L. oblata*. The possibility of spontaneous self-pollination was also excluded because none of the non-manipulated but bagged flowers set fruit (Fig. 6A and Table S2). Hence, *L. oblata* has a pollinator-dependent mixed mating system.

Dichogamy and herkogamy are the two main mechanisms preventing spontaneous self-fertilization [24, 27]. The fruit set and seed set for flowers subjected to manual self-pollination and a short overlap between male and female phases (Fig. 5B) suggested *L. oblata* has incomplete dichogamy [27]. The drastically reduced product of fruit set and seed set of flowers subjected to manual self-pollination (0.26%, Fig. 6C and Table S2) than that of geitonogamous pollination (5.04%, Fig. 6C and Table S2) could reflect the proterandry of this species. Because geitonogamy treatment demonstrated that this may be not only the result of genetic incompatibility, but also related to the low quantity and quality of available pollen when the stigma is receptive, as also reported for *Silene acutifolia* Link ex Rohrb. (Caryophyllaceae) [34]. and *Dianthus morisianus* Vals. (Caryophyllaceae) [35]. Despite being self-compatible and with a short overlap between pollen viability and stigma receptivity (Fig. 5B), spontaneous selfing did not occur in *L. oblata* (Fig.6 and Table S2) due to its reverse herkogamy (Fig. 2). This spatial segregation of sexual functions within the flower in *L. oblata* could reduce the contact between anthers and stigma [24, 36]. Hence, both incomplete dichogamy and reverse herkogamy affect the reproductive success in this threatened species, *L. oblata*.

### Conservation implications

*Lonicera oblata* experiences a harsh climate (eg., low temperature, snowfall, strong wind, and high dust conditions) during its flowering period. These factors may provide high pressure on the survival of *L. oblata* both intrinsic (e.g., reproductive system and genetic diversity) and external (e.g., the number and effective activity of pollinators). Based on our results, several conservation strategies are proposed. First, in situ conservation of all existing populations is required. Second, conservation of both habitat and insect diversity is highly recommended. *L. oblata* has a mixed mating system of outcrossing and pollinator-dependent self-fertilization, so enhancing pollination is crucial to maintain population genetic diversity and offspring fitness. Third, reintroduction and transplantation of seedlings from multiple sources is necessary to weaken intra-population inbreeding. Additionally, studies of seed biology and seedlings are required to examine appropriate strategies for promoting effective genetic exchange among populations.

## Conclusions

The reproductive biology of *L. oblata* was characterized. This endangered lithophytic species is endemic to cliff habitats in North China, and little was previously known about its reproduction. This species exhibits a suite of floral syndromes (e.g., bisexual and zygomorphic flowers, tubular and bilabiate corolla, brightly yellowish tepals, heavy-sweat fragrance, and sucrose-rich nectar) that adapt to insect-mediated cross-pollination. Comprehensive studies of OCI, P/O ratio, and artificial pollination suggested *L. oblata* had a highly pollinator-dependent mixed mating system of cross- and self-fertilization. Reverse herkogamy and incomplete dichogamy were observed to prevent autonomous self-pollination at the single flower level. Sweat bees contributed dominantly to the pollination success of *L. oblata* but they had a low visiting frequency. The combinations of low-flowering synchrony, asynchronous anthers dehiscence, and high duration of stigma receptivity allow successful reproduction in the harsh cliff environment. However, fragmented and limited numbers of populations and individuals, harsh climate and habitat environments, and pollen limitation threaten the survival of *L. oblata*. Finally, the results suggested several conservation strategies. This work not only illuminates the reproductive characteristics of an endangered species endemic to North China, but also provides insight into the reproductive characteristics of species endemic to limestone mountain habitats.

## Methods

### Study species and sites

*Lonicera oblata* is an early-spring flowering deciduous shrub up to 250 cm tall with opposite leaves (Fig. 1D). This species has densely or sparsely glandular hairs on its ranches, petioles, and peduncles. It has paired-flower inflorescences, and flowers are zygomorphic, hermaphrodite, paired, axillary, with a tubular two-lipped yellowish corolla (Figs. 1E and 2). The fruits are globose, with red fleshy berries (Fig. 1F), and seeds are brownish, suborbicular or ovoid-orbicular, and slightly compressed.

Our fieldwork was performed at Jiankou Great Wall (40°27′49″N, 116°29′39″E), Huairou District, Beijing, China. *L*. *oblata* grows near or on the top of the cliffs at an altitude of 800–950 m (Fig. 1B–C). The population consists of about 100 individuals and the canopy density is 0–65%. The other main coexisting plants in these habitats are trees of *Betula chinensis* Maxim. (Betulaceae), *Syringa pubescens* Turcz. (Oleaceae), and *Carpinus turczaninowii* Hance (Betulaceae); shrubs of *Myripnois dioica* Bunge (Asteraceae), *Rhamnus arguta* Maxim. (Rhamnaceae), *Zabelia biflora* (Turcz.) Makino (Caprifoliaceae), and *Spiraea trilobata* L. (Rosaceae); and herbs of *Chrysanthemum chanetii* H. Lév. (Asteraceae), *Potentilla simulatrix* Th. Wolf (Rosaceae), *Atractylodes lancea* (Thunb.) DC. (Asteraceae), and *O. rupifraga*. The fieldwork was carried out during the flowering and fruiting season (April to July) from 2016 to 2019.

The plant materials were carefully identified by the corresponding author of this article (Dr. Xianyun Mu), and a voucher specimen of *L. oblata* (collector and collection number: Yuanmi Wu, BJ_JK_XDQ01) was deposited in the Herbarium of Beijing Forestry University. Sample collection and field pollination experiments were permitted by the Bureau of Beijing Municipal Forestry and Parks. All plant materials used in this study were collected in compliance with local regulations.

### Floral traits and flower visitor observation

To determine the floral phenology, biology, and blooming process of *L. oblata*, flower buds of 5–10 randomly selected individuals were labeled and observed continuously until the flowers wilted for three consecutive years. The duration of pollen shed and changes of floral traits such as corolla morphology and stigma color were monitored and recorded. Additionally, 30 inflorescences in full blossom from 5 to 10 individuals were randomly selected, and the length of pedicel and flower, corolla length and diameter, floral tube length and width, and length of stigma and stamen were measured using a digital caliper (Pro’sKit PD-151).

The types and behaviors of flower visitors were monitored from 10:00 to 16:00 for seven consecutive sunny days in early May 2017 during peak flowering time. Flower visitors were recorded by photo and video (Nikon D7200) and captured with insect nets. The behavior and visiting frequency of flower visitors were recorded and compared. Tongue length and body width of captured insects were measured using a digital caliper, and compared with the floral tube length and width, respectively.

### Pollen viability and stigma receptivity

To assess the dynamic of pollen viability and stigma receptivity over different stages of flowering, we marked 32 fresh flower buds of *L. oblata*, and then bagged them to prevent pollen removal. Finally, different developmental stages (three different sizes of flower buds and five different flower opening days, with four flowers in each replicate) were collected at 10:00–12:00 for eight consecutive days (see Tables S1 for more details). Five anthers in each flower at different developmental stages of flowering were transferred to a centrifuge tube (2 mL) filled with 0.5% (g/mL) 2, 3, 5-triphenyl tetrazolium chloride (TTC) [19], thoroughly shaken to form a pollen suspension, and then placed in a water bath at 35 °C for 15 min. Some of the pollen suspension was placed on a microscope slide using a pipette, and the pollen grains were observed using an optical microscope (10 × 40 times, OLYMPUS BX51). Five fields were randomly selected from each slide to observe the staining status of pollen grains. If the pollen grains were dyed red, this indicated strong viability, light red indicated weak viability, and colorless pollens were inactive or sterile [20]. Following the criterion of Tong [37], the color change for pollen grains was classified into four grades (A, dark red; B, red; C, light red; D, not stained, Fig. S1) and corresponding weights (1.5, 1.0, 0.5, and 0) were assigned. Pollen viability was calculated as: Pollen viability = (([number of A pollen grains × 1.5] + [number of B pollen grains × 1.0] + [number of C pollen grains × 0.5] + [number of D pollen grains × 0]) / the total number of observed pollen grains) × 100%.

Stigmas at different developmental stages of flowering were collected and placed on a concave glass slide containing benzidine-hydrogen peroxide reaction solution (1% (g/mL) benzidine: 3% (g/mL) hydrogen peroxide: ddH_2_O = 4:11:22, volume ratio) [38]. The reaction (bubbles and color change) was observed and recorded under an optical microscope (10 × 10 times). We also recorded the time when bubbles appeared on the stigma. If the stigma was receptive, there would be a large number of bubbles and blue color surrounding the area of the stigma. Following the method of Tong [37], the stigma receptivity was measured by using assigned weights for the sum of the values obtained from four assessments: (a) bubbling start time × (− 0.1); (b) bubbling rate: 3, 2, 1 for fast, medium, and slow, respectively; (c) bubble number: 4, 3, 2, 1, 0 for bubble accumulation exceeding the stigma, bubble accumulation equal to the entire stigma, bubble accumulation in the middle of the stigma, bubble accumulation on the edge of the stigma, and no bubble accumulation, respectively; and (d) color change: 1or 0 for blue or not blue, respectively. A greater total value indicated stronger receptivity of the stigma.

### In vitro pollen germination and pollen longevity

Pollen grains were randomly collected from 36 fresh flowers of eight selected individuals and dried for 1 day in silica gel. In vitro pollen germination was tested after different times of storage (1–6 days, three repeats) to evaluate the pollen longevity of *L. oblata*. In vitro pollen germination was performed in a basic media consisting of 50 mg/L boric acid, 20% sucrose, 1% agar, and 100 mL distilled water, which was adjusted to pH 5.0. All media components were dissolved in boiling water and poured into petri dishes. Pollen grains were then applied to the surface of the cooled media and allowed to germinate for about 2 h in a dark environment. Five random views with at least 100 pollen grains per sample were examined under an optical microscope (10 × 10 times). A pollen grain was considered germinated when the pollen tube length exceeded the diameter of pollen grains. The pollen germination rate = the number of germinated pollen grains / the total number of pollen grains.

### Outcrossing index and pollen/ovule ratio

The outcrossing index (OCI) and the ratio of pollen grains to ovules (P/O) were determined to estimate the likelihood of pollination outcrossing (xenogamy) versus selfing (autogamy) based on the floral morphological characteristics [19, 20]. The OCI was estimated as the sum of the values obtained from: (a) flower size (0, 1, 2, 3 for corolla opening diameters of ≤1, 1–2, 2–6, > 6 mm, respectively); (b) herkogamy (1 or 0 for presence of herkogamy or not, respectively); and (c) dichogamy (1 or 0 for existence of protandry or homogamy, respectively) [19]. To calculate the P/O, 36 fresh flower buds were randomly collected from eight individuals. Anthers in each flower bud were transferred to a centrifuge tube (2 mL), filled with 15% (g/mL) glucose solution, and thoroughly shaken to form a pollen suspension. Next, a 5 μL subsample of the pollen suspension was transferred to a microscope slide using a pipette, and pollen grains were counted using an optical stereomicroscope (10 × 10 times). The total number of pollen grains = subsample number of pollen grains × volume of centrifuge tube / volume of pollen suspension in the pipette. We repeated this measurement 12 times for each flower bud, and then removed the maximum and minimum values before averaging. The total pollen number for each flower bud was recorded as P. The ovary of each flower bud was dissected using a blade, and the number of ovules was counted using an anatomic microscope (10 × 40 times, OLYMPUS SZX16) and recorded as O [20].

### Controlled pollination experiments

We randomly selected flowers from eight individuals to further evaluate the breeding system of *L. oblata*. Nine different types of pollination treatments were conducted yearly from 2016 to 2019 (Table S2): (1) apomixes test: flower buds were emasculated and covered with waxed paper bags, *N* = 66; (2) spontaneous self-pollination test: flower buds were covered with waxed paper bags, *N* = 54; (3) manual self-pollination test: flower buds were covered with waxed paper bags, and then flowers were manually pollinated with pollen from their own anthers and then rebagged, *N* = 46; (4) geitonogamous pollination test: flower buds were emasculated and covered with waxed paper bags, and then flowers were pollinated using pollen from other flowers of the same individual and then rebagged, *N* = 220; (5) xenogamous pollination test: flower buds were emasculated and covered with waxed paper bags, and then flowers were pollinated using pollen from different individuals before being rebagged, *N* = 216; (6) anemophilous pollination test: flower buds were emasculated and covered with 80-filter mesh bags, *N* = 172; (7) isolation animal pollination test: flower buds were covered with mesh bags with 80 mesh filter, *N* = 254; (8) pollinator-mediated cross-pollination test: flower buds were emasculated, *N* = 194; (9) natural pollination (control): flowers were marked and received no further manipulation, *N* = 595. All bags were removed after the flowers withered. Fruit set and seed set of each treatment were estimated in early July when fruits ripened.

### Data analysis

Datasets with a normal distribution were analyzed by Student’s t-test and one-way ANOVA. The lengths of stamens and pistils were compared using Student’s t-test. We used one-way ANOVA followed by a Tukey HSD test for multiple comparisons to test the differences of pollinator and floral dimensions, visit frequency and time, and fruit production under different pollination treatments. Kruskal-Wallis test was used to analyze the seed production under different pollination treatments because these datasets were not normally distributed. SPSS version 20.0 (IBM, Armonk, NY, USA) was used for data analysis, and all data were plotted with GGPLOT2 version 3.3.5 [39] in R version 4.0.0 (R Core Team, 2020).

## Supplementary Information

**Additional file 1: Figure S1.** Four grades of dyeing pollen of *Lonicera oblata* (A dark red; B red; C light red; D not dyed).

**Additional file 2: Table S1.** The pollen viability and stigma receptivity of *Lonicera oblata* during the different flowering periods.

**Additional file 3: Table S2.** The different treatment groups and results of pollination experiment of *Lonicera oblata*.

**Additional file 4: Video S1.** *Lasioglossum* sp. visits the flowers of *Lonicera oblata.*

## Data Availability

The datasets used and/or analyzed during the current study are available from the corresponding author on reasonable request.

## Abbreviations

OCI: The outcrossing index
P/O: The ratio of pollen grains to ovules
TTC: 2, 3, 5-triphenyl tetrazolium chloride
ANOVA: Analysis of variance
SD: Standard deviation
