# Supplementary figures and images for: Reproductive biology of an endangered lithophytic shrub and implications for its conservation
Source: BMC Plant Biol. 2022 Feb 22;22:80. doi: 10.1186/s12870-022-03466-3 (PMC8862588; doi:10.1186/s12870-022-03466-3)

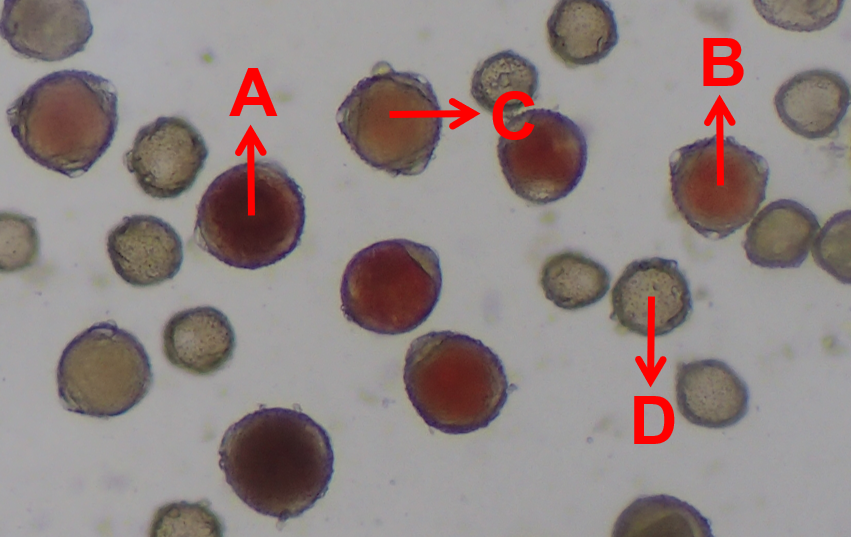

Supplement: Supplementary file 1 — Additional file 1: Figure S1. Four grades of dyeing pollen of Lonicera oblata (A dark red; B red; C light red; D not dyed). [file 12870_2022_3466_MOESM1_ESM.tif]
